# Temporal and spatial changes in bone mineral content and mechanical properties during breast-cancer bone metastases

**DOI:** 10.1016/j.bonr.2022.101597

**Published:** 2022-06-12

**Authors:** Anneke S.K. Verbruggen, Elan C. McCarthy, Roisin M. Dwyer, Laoise M. McNamara

**Affiliations:** a Mechanobiology and Medical Device Research group (MMDRG), Biomedical Engineering, College of Science and Engineering, National University of Ireland Galway, Ireland.; b Discipline of Surgery, Lambe Institute for Translational Research, National University of Ireland Galway, Ireland

**Keywords:** Bone, Mechanobiology, Metastasis, Nanoindentation, Computed tomography, Breast cancer

## Abstract

Cancer cells favour migration and metastasis to bone tissue for 70–80 % of advanced breast cancer patients and it has been proposed that bone tissue provides attractive physical properties that facilitate tumour invasion, resulting in osteolytic and or osteoblastic metastasis. However, it is not yet known how specific bone tissue composition is associated with tumour invasion. In particular, how compositional and nano-mechanical properties of bone tissue evolve during metastasis, and where in the bone they arise, may affect the overall aggressiveness of tumour invasion, but this is not well understood. The objective of this study is to develop an advanced understanding of temporal and spatial changes in nano-mechanical properties and composition of bone tissue during metastasis. Primary mammary tumours were induced by inoculation of immune-competent BALB/c mice with 4T1 breast cancer cells in the mammary fat pad local to the right femur. Microcomputed tomography and nanoindentation were conducted to quantify cortical and trabecular bone matrix mineralisation and nano-mechanical properties. Analysis was performed in proximal and distal femur regions (spatial analysis) of tumour-adjacent (ipsilateral) and contralateral femurs after 3 weeks and 6 weeks of tumour and metastasis development (temporal analysis). By 3 weeks post-inoculation there was no significant difference in bone volume fraction or nano-mechanical properties of bone tissue between the metastatic femora and healthy controls. However, early osteolysis was indicated by trabecular thinning in the distal and proximal trabecular compartment of tumour-bearing femora. Moreover, cortical thickness was significantly increased in the distal region, and the mean mineral density was significantly higher in cortical and trabecular bone tissue in both proximal and distal regions, of ipsilateral (tumour-bearing) femurs compared to healthy controls. By 6 weeks post-inoculation, overt osteolytic lesions were identified in all ipsilateral metastatic femora, but also in two of four contralateral femora of tumour-bearing mice. Bone volume fraction, cortical area, cortical and trabecular thickness were all significantly decreased in metastatic femora (both ipsilateral and contralateral). Trabecular bone tissue stiffness in the proximal femur decreased in the ipsilateral femurs compared to contralateral and control sites. Temporal and spatial analysis of bone nano-mechanical properties and mineralisation during breast cancer invasion reveals changes in bone tissue composition prior to and following overt metastatic osteolysis, local and distant from the primary tumour site. These changes may alter the mechanical environment of both the bone and tumour cells, and thereby play a role in perpetuating the cancer vicious cycle during breast cancer metastasis to bone tissue.

## Highlights

• Temporal and spatial analyses of bone tissue properties following breast cancer metastasis
• Trabecular thinning initiated by 3 weeks but overt osteolysis not evident until 6 weeks.
• Increased bone mineralisation and distal cortical thickness by 3-weeks post-inoculation

## 1 Introduction

Metastasis occurs when cancer cells migrate from a primary tumour site and colonise a secondary organ, and is the primary cause of mortality in cancer patients (Langley and Fidler, 2007, Weigelt et al., 2005). Cancer cells favour metastasis to bone tissue for 70–80 % of advanced breast cancer patients (Coleman and Rubens, 1987, Plunkett and Rubens, 1999) and can lead to bone destruction (osteolysis) or tissue formation by a process known as osteoblastic metastasis (Clines and Guise, 2005, Kozlow and Guise, 2005, Mundy, 2002). Metastatic invasion of the skeletal environment leads to severe pain, increased fracture risk, nerve compression and hypercalcemia (Clines and Guise, 2005, Kozlow and Guise, 2005). In healthy bone the coordinated activities of osteocytes, osteoblasts, and osteoclasts govern bone tissue structure and composition, and ensure a constant remodelling process in response to mechanical cues due to skeletal loading (McNamara, 2010, Mellon and Tanner, 2012). Paget's ‘Seed and Soil’ theory (1889) suggests that cancer cells migrate to bone tissue due to its easily manipulated remodelling process and attractive physical properties. Tumour cells first arrive within the bone marrow ECM, a mechanosensitive tissue that houses osteoblasts and osteoclasts and a source of mechanobiological cues for regular bone remodelling (Lynch et al., 2020), before tumour cells ultimately adhere to the bone tissue surface (Zheng et al., 2013; Allocca et al., 2019). During bone metastasis, invading tumour cells disrupt the normal bone remodelling process over time by releasing growth factors, most notably PTHrP, that activates osteoclasts to collaborate and resorb the bone matrix and releasing chemotactic stimuli and additional growth factors (TGFβ, Ca^2+^) (Yoneda et al., 1994; Guise, 2002; Kumar and Weaver, 2009). Growth factors and cytokines, stored within the bone extracellular matrix (ECM) and released upon resorption, are key attractants for invading breast cancer cells, and facilitate further tumour cell proliferation (Yoneda et al., 1994). This process of tumour cell proliferation, osteoclast resorption and osteoblastic metastasis is thereby perpetuated in a ‘vicious cycle’ of cancer (Clines and Guise, 2005).

To understand fracture susceptibility following metastasis, bone mineral density (BMD) analysis and mechanical assessment have been conducted to characterise bone tissue from patients with bone metastases (Kaneko et al., 2003, Nazarian et al., 2008). Micro-CT analyses of the femoral diaphysis of patients with mixed cancer metastases (lung, breast, prostate, 53–78 years old) revealed significantly decreased mean BMD in cadaveric cortical bone in patients with metastases (Kaneko et al., 2003). Mechanical tests were performed on patient cortical bone samples with metastatic lesions and compared to cancer-free bone regions (Kaneko et al., 2003), which revealed significantly lower Young's modulus, yield strength and ultimate strength under compression, as well as lower Young's modulus under tension. These findings were suggested to be a result of increased cortical bone ductility as osteolysis progresses (Kaneko et al., 2003). In a follow on study of distal femora of human metastatic patients (45–88 years old), no differences were reported in bone density or Young's modulus between groups, which was attributed to low patient numbers and large metastatic variation (Kaneko et al., 2004). A later study analysed bone cores of metastatic male and female patient bone tissue (lung, breast, prostate, ovarian, colon, 36–83 years old), sourced from proximal femurs and vertebrae either at surgery for fracture treatment or autopsy, and compared to site-matched cadaveric bone tissue of cancer-free patients (Nazarian et al., 2008). This study found significantly lower bone mineral content (by micro-CT analysis), decreased weighted average gray levels (via backscatter emission, BSE), decreased Young's modulus and compressive yield strength, and also decreased Young's modulus and hardness, via dry nanoindentation, in metastatic bone tissue compared to healthy samples (Nazarian et al., 2008). Although these studies established that osteolytic cancer metastasis is associated with a decrease in human bone mineral content and mechanical properties, it remains that patient variation arises due to differences in age, extent of metastasis, underlying conditions (e.g. osteoporosis) and treatment regimens (e.g. chemotherapy) (Yao et al., 2020). Such variability has limited a comprehensive understanding of changes in bone tissue during metastasis.

Pre-clinical animal models have enabled the study of changes in bone tissue composition and nanoscale mechanical (nano-mechanical) properties after metastasis by breast cancer cells. Three weeks following intracardiac inoculation of HeLa cervical cancer cells in female athymic rnu/rnu rats (5–6 weeks old) trabecular vertebral bone tissue presented osteolytic bone lesions of decreased crystallinity, crystal size and collagen quality as detected by Raman Spectroscopy (Burke et al., 2016). Notably, High performance Liquid Chromatography (HPLC) analysis revealed a significant increase in AGE collagen crosslink pentosidine compared to healthy controls, previously associated with increased risk of fracture failure (Burke et al., 2016). A similar study also found decreased crystal width (Burke et al., 2017), whereas another showed decreased bone mineral density, trabecular thickness, number and bone volume in rat vertebral bone tissue using micro-CT analysis (7.4 μm), 3 weeks post-intracardiac inoculation of HeLa cells (Burke et al., 2018). Another animal study directly injected MDA-MD-231 derived F10 breast cancer cells into the intercondylar fossa of the right femurs of 8-week-old female NCr nude mice and compared to the left femurs of these animals receiving SHAM injections of culture medium as internal controls (Arrington et al., 2006). This study reported osteolytic lesions in 58 % of animals by 3 weeks post tumour injection via radiography, but no significant differences in bone strength via whole bone torsion testing were reported, whereas dual-energy X-ray absorptiometry (DEXA) measurements of areal BMD had significantly increased in both contralateral femora (internal controls) and tumour-injected femora by 3 weeks (Arrington et al., 2006). By 6 weeks tumour-injected femora with osetolytic lesions had significantly lower areal BMD compared to those with no detectable lesions and to controls. Meanwhile, bone stiffness had significantly decreased in tumour injected femora, both with or without lesions, compared to contralateral femurs, and most animals did not reach the 9 week time point due to high risk of fracture (Arrington et al., 2006). In a later study osteolytic destruction was visible within 3 weeks after direct innocculation of MDA-MD-231 cells into distal femora of nude NCr mice, yet no changes in BMD were detected (micro-CT at 12 μm resolution) but by 6 weeks post-injection there was a significant reduction in BMD compared to contralateral limbs that did not receive injections (Arrington et al., 2008). A similar study involved reconstructing the tumour-bearing and contralateral mouse tibiae in silico, using finite element analysis, and subjected to 3-point bend testing (Mann et al., 2008). This study separated cohorts according to osteolysis severity rather than time points. Interestingly, a strong corrolation between bone density and corresponding mechanical properties was found, specifically in bone tissue stiffness and strength, and corrolations were greatest in micro-CT derived densities as opposed to DEXA imaging (Mann et al., 2008). In another pre-clinical study, distal tibiae were analysed from athymic BALB/c mice (4 weeks old) after intravenous injection with MDA-MB-231 breast cancer cells. By 32 days after intravenous injection both trabecular bone mineral content (via histomorphometry) and cortical bone elastic modulus, determined by atomic force microscopy (AFM), were significantly reduced (Richert et al., 2015). Another study reported significantly decreased dry nanoindentation modulus in femur diaphysis cortical bone samples by 14-days after intracardiac injection of osteolytic B16F10 melanoma cells into C57BL/6 female mice (Sekita et al., 2017). Interestingly, a comparative study of young (6 weeks) and mature (16 weeks) nude BALB/c mice that received intracardiac injections of MDA-MB-231 cells, demonstrated that the rate of osteolytic lesion development at 3 weeks was greatly increased in young mouse bone expressing higher rates of metabolic activity (Wang et al., 2015). A recent animal study performed daily intraperitoneal injections of MDA-MB-231 tumour-conditioned media into nude BALB/c mice for 3 weeks (Chiou et al., 2021), while also introducing mineral-binding dyes green calcein (at day 13) and xylenol orange (day 20) into the bone marrow cavity of the proximal tibiae. They reported an increased rate of bone mineral apposition in the endosteal cortical bone tissue, adjacent to the growth plate, within 7 days of early metastatic development, with no significant changes in rate of trabecular bone mineral apposition (Chiou et al., 2021). Interestingly, micro-CT analysis at 3 weeks of this study found significantly increased cortical and trabecular bone volume fraction and thickness compared to healthy controls (Chiou et al., 2021). The above studies characterise changes in bone mineral content and mechanical properties, and their timeline, upon overt osteolytic destruction of the bone tissue microenvironment. However, how these bone tissue material properties compare prior to and following the development of breast cancer osteolytic lesions is not fully understood. Furthermore, these animal studies involved inoculation of cancer cells directly into the femoral cavity or peripherally via intra-cardiac, intraperitoneal, or intravenous sites. Such approaches only partially recapitulate the breast cancer metastatic process in vivo, as they do not capture cancer cell extravasation from the primary tumour and homing to the bone environment (Kretschmann and Welm, 2012), which would dictate the timing of adhesion and colonisation of the bone. Furthermore, these studies involved immunocompromised (athymic or nude) animal models, albeit that the immune system may play an important role in tumour–bone cellular interactions during the metastatic process (Kretschmann and Welm, 2012).

One study investigated bone tissue after MDA-MB-231 breast cancer cells were injected into the mammary fat pads of female BALB/c mice, and confirmed via bioluminescent imaging that metastatic cells were present in the trabecular bone region of the proximal tibias 7 weeks post-inoculation (He et al., 2017). Moreover, in this study X-ray scattering analysis revealed significantly shorter HA crystals and large-area Raman imaging demonstrated decreased mineral crystallinity, in the tibiae of these mammary-inoculated mice when compared to healthy controls at this 7-week time point, which was proposed to indicate immature bone mineral (He et al., 2017). Inoculation of triple-negative 4T1 cells into the mammary pad of BALB/c immunocompetent mice results in primary tumour formation within one week post-inoculation and has a reported 100 % incidence of metastasis to bone tissue 3–4 weeks post-inoculation, confirmed by H&E histological staining (Lelekakis et al., 1999; Yoneda et al., 2000). This 4T1-BALB/c mouse model recapitulates key steps of in vivo breast cancer metastasis from a primary tumour site to bone, whereby breast cancer cells which have intravasated into the capillary network subsequently extravasate to the bone marrow niche to initiate colonisation and the metastatic process (Kretschmann and Welm, 2012; Allocca et al., 2019). This animal model has enabled the study of biochemical treatments such as kinase inhibitors (A77636, PD407824, pitavastatin) for impeding the development of osteolytic lesions upon breast cancer metastasis (Jiang et al., 2018, Minami et al., 2017, Wang et al., 2019). However, the time-dependant and spatial evolution of bone tissue properties following primary tumour development has not been fully characterised. In particular, the temporal changes in bone physical properties between cancer cell extravasation and subsequent overt osteolytic destruction have not yet been investigated. These changes may alter the tumour-adjacent and non-tumour-bearing mechanical environments of bone and tumour cells over time, and might thereby play a role in perpetuating the cancer vicious cycle and tumour invasiveness during breast cancer metastasis to bone.

The objective of this study was to investigate changes in bone mass and microarchitecture, mineral content and nano-mechanical properties of bone tissue that arise upon breast cancer metastatic cell invasion, by high-resolution micro-CT imaging and nanoindentation analysis of bone tissue from an immunocompetent BALB/c mouse model inoculated with 4T1 breast cancer cells in the mammary fat pad, and relate these findings to the temporal development and location of the primary tumour mass.

## 2 Methods

### 2.1 Animal model

The current study utilises the 4T1-BALB/c animal model, both for its high metastatic rate and its ability to recapitulate the in vivo development of breast cancer metastasis from a primary tumour site to bone (Kretschmann and Welm, 2012). This research was conducted with approval from the Animal Care Research Ethics Committee (ACREC) at the National University of Ireland in Galway, and the Health Products Regulatory Authority (HPRA), the national authority for scientific animal protection in Ireland.

Female BALB/c immunocompetent mice (6 weeks old) were inoculated with 1 × 10^5^ 4T1 murine breast cancer cells via direct injection into the surgically exposed right 4th inguinal mammary fat pad, hereon referred to as the metastatic ipsilateral side (MET-IPS). These cells were previously transduced to express the luciferase gene, for downstream bioluminescent imaging. Femurs were also collected from the contralateral side (MET-CONTRA) to investigate and compare spatial changes in the bone tissue of non tumour-bearing femurs. The mice were maintained under normal laboratory conditions with food and water provided ad libitum. Healthy control models (CTRL) were sex-, strain- and age-matched, maintained under identical conditions but did not receive inoculations. The first animal cohorts were euthanised at 3 weeks post-inoculation (CTRL *n* = 5, MET-IPS n = 5, MET-CONTRA n = 5) and a second cohort had an endpoint of 6 weeks post-inoculation (CTRL *n* = 6, MET-IPS *n* = 7, MET-CONTRA n = 7). The 3 week timepoint was chosen because H&E staining confirmed metastatic tumour cell presence in femur trabecular bone tissue just 19 days following inoculation of 4T1 breast cancer cells into the mammary fat pad of BALB/c mice (Lelekakis et al., 1999), whereas 6 weeks was sufficient time for overt osteolytic lesions to develop (Arrington et al., 2006; Mann et al., 2008; Wang et al., 2015; Chiou et al., 2021). Although all left and right femurs of each control mouse were available for analysis (*n* = 12), a large sample size in the control group would exaggerate tendency to reject the null hypotheses (Faber and Fonseca, 2014). Therefore, control sample sizes similar to metastatic sample sizes were analysed. Bioluminescent imaging was performed at the conclusion of the 6 week cohort study to visualise disease progression. Animals received an intraperitoneal injection of D-luciferin (150 mg/kg), suspended in 150 μl Dulbecco's Modified Phosphate Buffered Saline (DPBS), followed by imaging under inhalation anaesthesia (1–2 % isoflurane) using an IVIS® Lumina LT (Perkin Elmer, USA). All femurs were harvested, muscle and tendon soft tissues removed, wrapped in PBS-soaked gauze and stored at −20 °C.

### 2.2 Micro-computed tomography

**Fig. 1:**
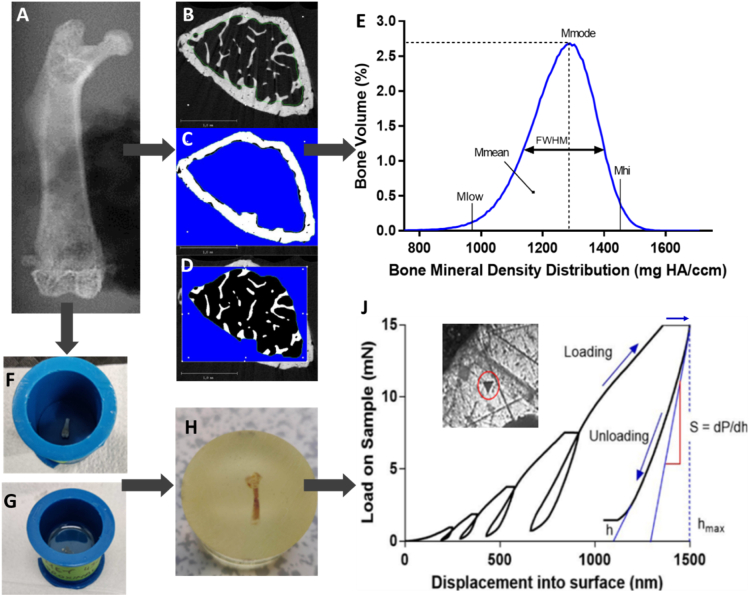
Sequence of sample analyses of a single BALB/c mouse femur. (A) Scout view prior to micro-CT scanning, (B) single distal femur greyvalue micro-CT scan, with (C) cortical and (D) trabecular bone region contours isolated and evaluated to generate each (E) bone mineral density distribution (BMDD) curve, including M_low_, M_med_, M_hi_, FWHM, M_mode_ and M_mean_ parameters of bone mineralisation. (F) Femur sample, sectioned with a low speed saw and (G) covered in epoxy resin which is then (H) smooth polished to expose cortical and trabecular surface regions in preparation for (J) nanoindentation, using Oliver and Pharr equations (1992) tests into the bone surface (inset) used to calculate nano-mechanical properties.

Micro-computed tomography (micro-CT) is a rapid, non-destructive analytical technique used for detailed quantification of bone mineral density throughout chosen volumes of interest (VOIs), and incorporates the use of hydroxyapatite phantoms to allow for analysis of bone tissue mineralisation (Bouxsein et al., 2010). Samples were thawed overnight at 4 °C, placed in 9 mm diameter chambers and immersed in phosphate buffered saline (PBS) at room temperature in a static, upright position during the imaging process. Two separate VOIs, at the proximal and distal metaphysis, were scanned to investigate cortical and trabecular bone mineral at locations local and distant from the tumour site. The proximal VOI was defined as spanning from the most proximal point of the femoral head to 4 mm in the distal direction. The distal VOI was chosen 0.25 mm from the distal growth plate, to avoid mineral variation effects in that region, and spanned 2 mm in length along the femoral shaft in the proximal direction. Quality calibrations were conducted weekly, which involved scanning a phantom of known mineral densities (0, 100, 200, 400, 800 mgHA/cm^3^). High resolution scans were taken at a voxel size of 5μm^3^, suitable for detecting changes in mouse femur trabecular thickness (40–60 μm). The following parameters were applied: 70kVp peak X-ray tube voltage, 57 μA tube current, 900 ms integration time, frame averaging of 5, 0.8 Gaussian filter and Support value of 1, Scanco Medial μCT100. A 0.5 ml aluminium filter was used to reduce the effects of beam hardening. A global density threshold of 513.7 mg HA/cm^3^ (3000 HU) captured both cortical and trabecular tissue, while eliminating soft tissues such as tendon or muscle fibers (Ravoori et al., 2010). A single global threshold value for all regions of interest was applied to eliminate compounding factors (Bouxsein et al., 2010). In sample scans where femoral bone features (femoral head, greater and lesser trochanters) were entirely absent, only scans where bone tissue was present were analysed. Cortical bone outlines were isolated using Scanco Medical (USA) integrated automated algorithms, while the trabecular region was manually delineated as standard (Bouxsein et al., 2010) (Fig. 1B-D), maintaining approximately 50 μm of space between the contour line and cortical bone internal edge. VOIs were reconstructed as 3D models and evaluation scripts, developed by Scanco Medical and generated using Image Processing Language (IPL), were applied to determine mineralisation parameters. Bone volume fraction (BV/TV), cortical and trabecular thickness (Ct.Th, Tb.Th), trabecular number (Tb.N) and trabecular separation (Tb.Sp) are indicators of bone loss and decreased vascularisation (Zeitoun et al., 2019). IPL was also used to determine mineral density ranges below, above and between the 25th and 75th percentiles (M_low_, M_high_, M_med_), most frequent mineral density value (M_mode_), Structure Model Index (SMI) which indicates trabecular shape (value range from 0 = flat plate, to 3 = cylindrical) and a heterogeneity indicator, which was based on the full width at half maximum (FWHM) of the bone mineral density distribution (BMDD) curve, see Fig. 1E. Weighted mean bone mineral density (M_mean_), defined as the average density value weighed according to frequency, was calculated using the following equation (Roschger et al., 2008; O’Sullivan et al., 2020).

(1) $$M_{\mathrm{mean}}=\sum \frac{x_{i}x\;{\mathrm{freq}}_{i}}{100}\;$$

### 2.3 Nanoindentation

Nanoscale mechanical testing was performed to evaluate the effect of metastatic invasion and tumour presence on cortical and trabecular bone tissue elastic modulus and hardness, and this approach is widely used in human and murine studies (Lane et al., 2006; Tang et al., 2007; Nazarian et al., 2008; Sekita et al., 2017; Burke et al., 2017). Immediately following micro-CT scanning, femurs were sectioned in half, using a low speed saw (Buehler, Germany) and centrifuged to eject bone marrow (Amend et al., 2016), before dehydrating in ascending concentrations of dH_2_O-diluted pure ethanol (50 %, 70 %, 80 %, 90 %, 100 %, 100 %) at 4 °C for 5 min intervals. Samples were embedded in a 2:1 ratio mixture of Epothin 2 epoxy resin and hardener (Buehler, Germany), vacuumed to eliminate trapped air and allowed to harden at room temperature over 72 h (Fig. 1-F, 1-G). Embedded samples (Buehler) were polished with diamond suspensions (9 μm, 3 μm, 1 μm, 0.05 μm) to expose proximal and distal femur halves for indentation testing in the transverse direction (Fig. 1-H). All mechanical testing of samples was performed within 2 months of the embedding process to avoid the long-term impact of epoxy resin on the nano-mechanical properties of bone tissue under nanoindentation (Mittra et al., 2006). A nanoindenter (G200, Keysight Technologies, USA), equipped with an Accutip Berkovich diamond indenter (ISO1518) was used for testing, with calibration performed on a standard fused silica sample (Corning 7980) to establish a relationship between the contact area and indenter depth. For each sample region of interest, at least 10 dry nanoindentation tests (and a maximum of 16 indents) were performed and averaged for cortical bone surfaces, and separately indented and averaged for trabecular bone surfaces. Cortical regions were indented with equidistant spacing of 40 μm to avoid interference, while trabecular locations were manually selected >50 μm from each indent and at least 10 μm from the trabecular edge to avoid any influence of surrounding epoxy resin (Rho et al., 1997; Hay et al., 2000). Locations for proximal and distal region testing were selected for sufficient surface area to consistently perform at least 10 indents per bone region, while allowing for adequate spacing between indents and the epoxy resin barrier. To consider potential differences in areas near to osteolytic tumour involvement, we indented tissue in the proximal sub-region of interest on the medial (tumour) side of the femoral neck. To consider areas not adjacent to tumour tissue, a distal region on the medial side was studied, and both tumour-bearing and non-tumour-bearing femurs analysed. A 5-cycle loading regime, at 10 nm/s loading rate and maximum load of 15mN, was applied (Fig. 1-J). A peak hold time of 30s was included in each cycle and environmental conditions were accounted for by performing these tests within a sealed chamber. Thermal drift effect was reduced in two ways: a) Test initiation was delayed until this measurement reached 0.1 nm/s or lower and b) the indenter was unloaded to 10 % load (1.5mN) and thermal drift recorded for 90s, then Young's modulus and hardness calculations adjusted accordingly (Nanosuite software, Keysight Technologies, USA). Nanoindentation equations demonstrated by Oliver and Pharr (1992) were used to first determine contact stiffness, *S*, as the slope of the final unloading curve of each 5-cycle test, using:

(2) $$S={\left(\frac{\mathrm{dP}}{\mathrm{dh}}\right|}_{h_{\max}}$$

where maximum load, *P*, reached indentation depth, *h*. Substituting in the above contact stiffness and applying projected contact area, *A*, reduced modulus *E*_*r*_ is given by:

(3) $$E_{r}=\frac{\sqrt{\pi }}{2}\frac{S}{\sqrt{A}}$$

With known values of Berkovich indenter Young's modulus (1141 GPa) and Poisson's ratio (0.07), a Poisson's ratio of bone was assumed to be the value of 0.3 as standard, (Silva et al., 2004; Miller et al., 2007; Tang et al., 2007) and the Young's modulus *E* of the indented bone tissue was calculated (Eq. (3)). Finally, bone tissue contact hardness was quantified (Eq. (4)).

(4) $$\;E=\left(1-v^{2}\right){\left[\frac{1}{E_{r}}-\frac{\left(1-{v_{i}}^{2}\right)}{E_{i}}\right]}^{-1}$$

(5) $$H=\frac{P}{A}$$

### 2.4 Statistical analysis

Statistical analyses were performed using MiniTab (version 17) software with femur samples analysed from each group (CTRL, MET-IPS, MET-CONTRA) from each cohort (3 weeks, 6 weeks). Each parameter was confirmed for normal distribution in these groups (Kolmogorov-Smirnov test) and assessed for equal variance (F test). Student *t*-tests were implemented to determine whether averaged data was statistically significant between groups of equal variance, and Welsh's test applied where sample groups had unequal variance. Statistical outliers were identified via Grubb's test and were not rejected due to rarity and the natural variation expected in results between individual mouse bone geometries. The results are displayed as mean ± standard deviation, with significance defined as a *p* value of <0.05, and greater significance (*p* < 0.01, *p* < 0.001) also indicated.

## 3 Results

### 3.1 Primary tumour development and disease burden

**Fig. 2:**
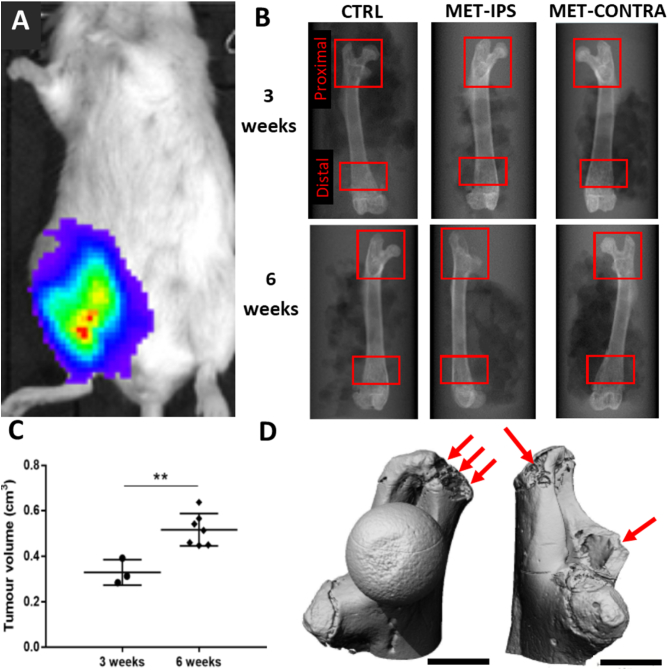
Tumour development and osteolytic destruction. (A) IVIS scan of BALB/c mouse, with 4T1 breast cancer cells populating greatest in regions highlighted in red according to the coloured contour. (B) Scout views of whole femurs from each disease group at 3- and 6-weeks post-inoculation of 4T1 breast cancer cells, including right-sided controls, with volumes of interest indicated (red boxes). (C) Measured tumour volumes at 3 weeks and 6 weeks post-inoculation (D) Two 3D reconstructions of MET-IPS proximal femurs at 6 weeks post-inoculation, with metastatic osteolytic destruction indicated (red arrows), scalebars 1 mm.

**Fig. 3:**
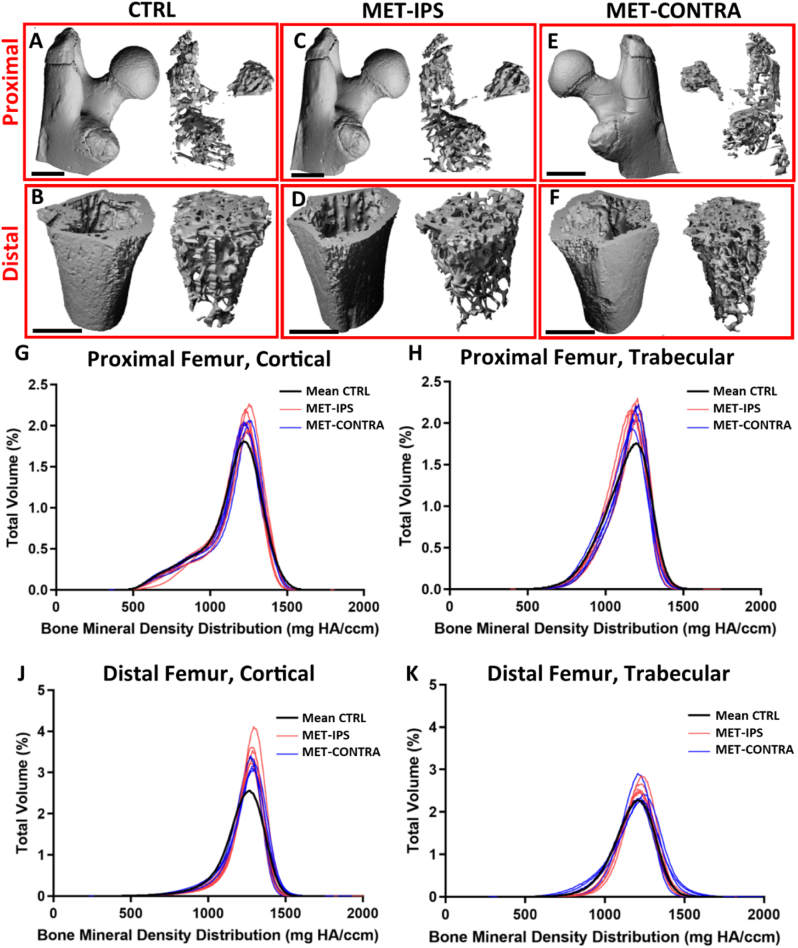
BALB/c disease and control mouse femurs at 3 weeks post-inoculation. 3D reconstructions of (A,C,E) proximal VOIs and (B,D,F) distal VOIs of a femur from each disease group, with contour isolation of (left) cortical and (right) trabecular bone regions. (G-K) Bone mineral density distribution (BMDD) curves in proximal and distal, cortical and trabecular regions of interest in BALB/c mouse femur VOIs at 3 weeks post-inoculation (CTRL *n* = 7, MET-IPS *n* = 5, MET-CONTRA n = 5).

At 3 weeks post-inoculation of 4T1 breast cancer cells into the mammary fat pad animal models presented with mammary tumours, visible with the un-aided eye as subcutaneous protrusions formed within 1 cm of the femoral head, and continued to grow up to 6-weeks. The volume of these tumours was 0.328 ± 0.046 cm^3^ for the cohort euthanised at 3 weeks and increased significantly to 0.515 ± 0.066 cm^3^ for those euthanised at 6 weeks (*p* = 0.004) (Fig. 2-C) and all masses at 6 weeks expressed luciferase visualised using IVIS imaging. For both cohorts, heavy disease burden was evident whereby softer organs, including the lungs, reported to contain circulating tumour cells in all disease animal models, indicating the initiation of the metastatic phase (Fig. 2-A). However, no overt osteolytic lesions were detectable using micro-CT scanning in the 3-week cohort (Fig. 3A-F). During sample collection, it was noted tumour masses remained outside the femoral head and were not in direct contact with bone tissue.

### 3.2 Osteolysis and bone loss (3 weeks post-inoculation)

**Fig. 4:**
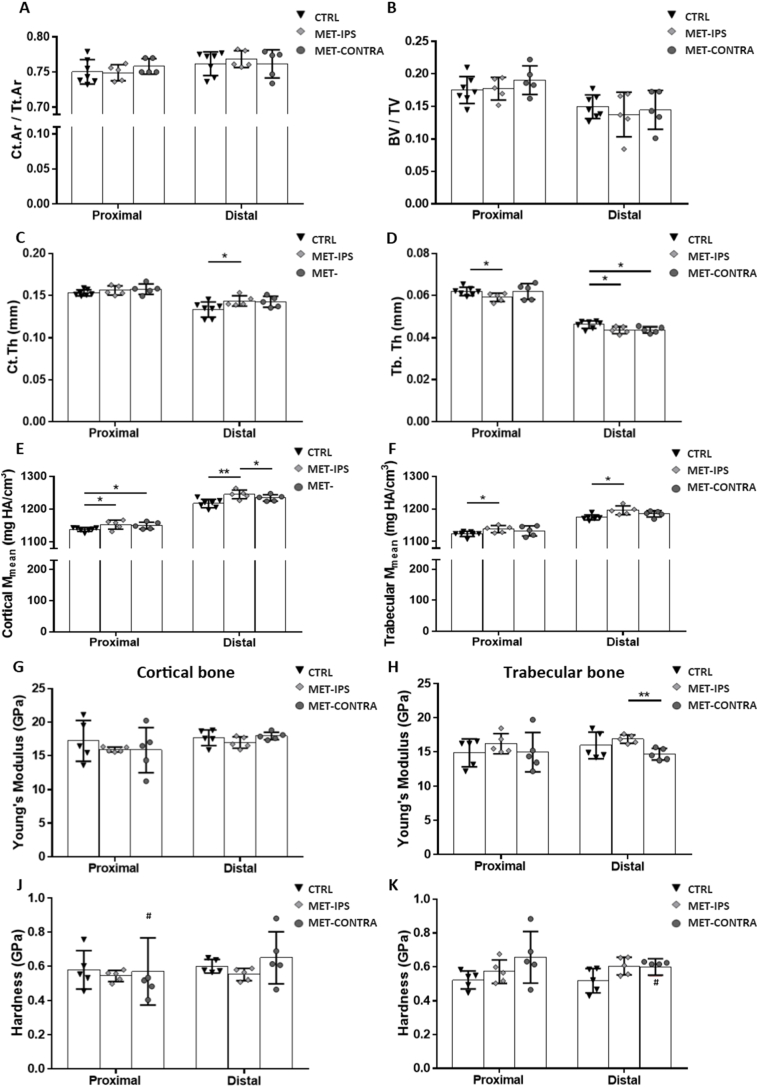
Mean parameters acquired from analyses of BALB/c mouse femurs 3 weeks post-inoculation. (A-F) Bone area fraction (Ct. Ar/Tt.Ar), bone volume fraction (BV/TV) cortical tissue thickness (Ct.Th), trabecular tissue thickness (Tb.Th), and mean mineral density (M_mean_) values acquired from micro-CT and BMDD analysis of proximal and distal VOIs in delineated cortical and trabecular bone regions. (G, H) Young's modulus and (J, K) hardness of cortical and trabecular bone tissue, obtained from nanoindentation tests in each bone region (CTRL *n* = 7, IPS *n* = 5, CONTRA n = 5). **#** Outlier, *p < 0.05, **p < 0.01.

After 3 weeks of 4T1 primary breast cancer development, micro-CT analysis of the BALB/c mouse femurs revealed that overt osteolysis had not yet been established and there was no significant difference in bone tissue area fraction (Ct.Ar/Tt.Ar) or volume fraction (BV/TV) in proximal or distal regions of MET-IPS and MET-CONTRA femora, when compared to healthy mouse femurs (CTRL), see Fig. 4-A, Fig. 4-B. However, trabecular thickness (Tb.Th) was significantly lower in the proximal femur regions of MET-IPS samples (0.059 ± 0.002 mm) compared to CTRL (0.062 ± 0.002 mm, *p* = 0.024) (Fig. 4-C, 4-D). In the distal femur regions, Tb.Th was also significantly lower both in MET-IPS (0.044 ± 0.001 mm) and MET-CONTRA (0.044 ± 0.001 mm) compared to CTRL samples (0.046 ± 0.002 mm) (*p* < 0.05).

### 3.3 Early changes in bone mineralisation in the proximal femur (3 weeks post-inoculation)

Bone mineral density distribution (BMDD) evaluations were conducted on micro-CT data from each animal after 3 weeks (Fig. 3G-3K). No significant difference in mineral distribution (M_low_, M_high_, M_med_), or homogeneity (FWHM) were detected between disease groups (MET-IPS and MET-CONTRA) in proximal femora when compared to healthy controls at 3 weeks, see Table 1, Fig. 3. In the proximal femur, local to the primary tumour, cortical and trabecular weighted mean density (M_mean_) were significantly higher in MET-IPS and MET-CONTRA samples compared to CTRL femurs (Fig. 4E, F). While but no differences in trabecular bone mode mineral density (M_mode_) were detected between MET-IPS and CTRL proximal femurs (*p* = 0.239), the MET-CONTRA samples were significantly higher in Mmode (1197.19 ± 14.10 mg HA/cm^3^) when compared to CTRL samples (1177.78 ± 4.33 mg HA/cm^3^, *p* < 0.05) (Table 1).

**Table 1:** Bone mineral content of healthy and disease mouse femurs 3 weeks post-inoculation (mean ± standard deviation) (CTRL *n* = 7, MET-IPS *n* = 5, MET-CONTRA n = 5) *p < 0.05, **p < 0.01, ***p < 0.001 relative to CTRL.

		Proximal Femur			Distal Femur		
	Units	CTRL	MET-IPS	MET-CONTRA	CTRL	MET-IPS	MET-CONTRA
Cortical bone							
Tt. Ar	mm^2^	1.39 ± 0.06	1.48 ± 0.07	1.41 ± 0.14	0.55 ± 0.03	0.55 ± 0.03	0.58 ± 0.04
Ct. Ar	mm^2^	1.05 ± 0.05	1.11 ± 0.06	1.07 ± 0.11	0.42 ± 0.02	0.42 ± 0.02	0.44 ± 0.03
Ct.Ar/Tt.Ar	*–*	0.75 ± 0.02	0.75 ± 0.01	0.76 ± 0.01	0.76 ± 0.02	0.77 ± 0.01	0.76 ± 0.02
Ct.Th	mm	0.1530 ± 0.003	0.1559 ± 0.005	0.1575 ± 0.01	0.1332 ± 0.008	0.1434 ± 0.006*	0.1424 ± 0.006
M_mean_	mg HA/cm^3^	1137.21 ± 5.94	1152.44 ± 12.49*	1149.41 ± 9.50*	1216.93 ± 12.10	1245.9 ± 12.09**	1234.07 ± 9.64*
M_mode_	mg HA/cm^3^	1223.41 ± 6.40	1234.12 ± 13.93	1228.67 ± 12.48	1268.17 ± 10.38	1284.68 ± 7.23*	1284.28 ± 10.40*
M low	%	4.17 ± 1.00	3.04 ± 0.85	2.74 ± 1.09	0.42 ± 0.11	0.37 ± 0.10	0.36 ± 0.07
M medium	%	89.01 ± 5.58	86.23 ± 6.79	84.57 ± 5.80	90.38 ± 7.81	92.29 ± 7.79	95.20 ± 3.68
M high	%	6.82 ± 6.19	10.73 ± 7.40	12.69 ± 6.15	9.20 ± 7.88	7.34 ± 7.86	4.43 ± 3.71
FWHM	mg HA/cm^3^	253.64 ± 21.87	238.49 ± 9.86	242.69 ± 17.86	196.52 ± 8.93	191.01 ± 12.05	199.28 ± 14.33

Trabecular bone							
TV	mm^3^	2.76 ± 0.18	2.92 ± 0.18	2.84 ± 0.19	2.60 ± 0.15	2.64 ± 0.14	2.71 ± 0.24
BV	mm^3^	0.48 ± 0.04	0.52 ± 0.06	0.54 ± 0.06	0.39 ± 0.06	0.37 ± 0.09	0.40 ± 0.10
BV/TV	*–*	0.18 ± 0.02	0.18 ± 0.02	0.19 ± 0.02	0.15 ± 0.02	0.14 ± 0.03	0.14 ± 0.03
M_mean_	mg HA/cm^3^	1122.97 ± 7.30	1137.80 ± 9.65*	1132.10 ± 13.92	1173.73 ± 7.39	1195.8 ± 12.15*	1184.75 ± 8.82
M_mode_	mg HA/cm^3^	1177.78 ± 5.25	1189.43 ± 21.60	1197.19 ± 14.10*	1201.12 ± 10.31	1224.24 ± 5.65**	1218.27 ± 16.92
Conn.D.	mg HA/cm^3^	102.72 ± 15.35	118.35 ± 12.52	122.64 ± 11.88	230.52 ± 27.02	217.19 ± 40.76	228.24 ± 47.58
SMI	*–*	0.70 ± 0.12	0.68 ± 0.10	0.57 ± 0.12	1.44 ± 0.15	1.57 ± 0.32	1.44 ± 0.27
Tb.N	1/mm	2.42 ± 0.24	2.48 ± 0.16	2.59 ± 0.15	4.04 ± 0.24	4.11 ± 0.44	4.19 ± 0.42
Tb.Th	mm	0.0619 ± 0.002	0.0591 ± 0.002 *	0.0619 ± 0.003	0.0462 ± 0.002	0.0436 ± 0.001*	0.0437 ± 0.001*
Tb.Sp	mm	0.438 ± 0.05	0.419 ± 0.03	0.410 ± 0.02-	0.239 ± 0.02	0.234 ± 0.03	0.231 ± 0.03
M low	%	1.03 ± 1.25	0.43 ± 0.16	0.50 ± 0.31	0.22 ± 0.12	0.19 ± 0.08	0.38 ± 0.31
M medium	%	93.01 ± 3.18	90.99 ± 5.59	92.37 ± 3.02	96.40 ± 2.69	95.95 ± 1.72	94.66 ± 6.10
M high	%	5.96 ± 3.88	8.58 ± 5.71	7.09 ± 3.19	3.37 ± 2.75	3.87 ± 1.76	4.96 ± 6.26
FWHM	mg HA/cm^3^	258.14 ± 50.24	241.51 ± 29.13	245.07 ± 20.30	249.65 ± 32.47	251.34 ± 17.63	261.82 ± 29.01

### 3.4 Early changes in bone mineralisation and increased cortical thickness in the distal femurs (3 weeks post-inoculation)

From micro-CT analyses of the distal femurs at 3 weeks, no significant difference in mineral distribution (M_low_, M_high_, M_med_), or homogeneity (FWHM) were detected between disease groups (MET-IPS and MET-CONTRA) compared to healthy CTRL samples, see Table 1. Cortical bone M_mean_ and M_mode_ were significantly higher in both the MET-IPS and MET-CONTRA disease groups when compared to CTRL group (Table 1). Similarly, in the distal trabecular region, M_mean_ and M_mode_ were significantly higher in the MET-IPS group compared to CTRL samples (*p* < 0.05, *p* < 0.001) (Table 1). Interestingly, though not statistically significant, a trend of increasing M_mean_ in distal trabecular MET-CONTRA samples (1184.75 ± 8.82 mg HA/cm^3^) is notable when compared to CTRL samples (1173.72 ± 7.39 mg HA/cm^3^) (*p* = 0.058). Distal femur Ct.Th was also significantly higher in MET-IPS samples (0.143 ± 0.006 mm) when compared to CTRL samples (0.133 ± 0.008 mm).

### 3.5 Cortical bone stiffness reduced in distal ipsilateral femurs compared to contralateral side (3 weeks post-inoculation)

At 3 weeks, in the distal region, MET-IPS femora trabecular bone had significantly higher Young's modulus (16.88 ± 0.55 GPa) when compared to MET-CONTRA femurs (14.67 ± 0.76 GPa, *p* = 0.002) (Fig. 4-H) but did not differ compared to CTRL (15.94 ± 1.75 GPa, *p* = 0.219). Aside from this result, no differences were seen in Young's modulus or hardness values in any other regions analysed at 3 weeks (Table 2, Fig. 4-G, J, K).

**Table 2:** Young's modulus and hardness (mean ± standard deviation) at 3 weeks post-injection of breast cancer cells, obtained from nanoindentation mechanical tests in each region and bone tissue type of each femur. Includes mean ± standard deviation (CTRL *n* = 7, MET-IPS *n* = 5, MET-CONTRA n = 5). **††***p* < 0.01, relative to MET-IPS.

	Proximal Femur			Distal Femur		
	CTRL	MET-IPS	MET-CONTRA	CTRL	MET-IPS	MET-CONTRA
Cortical bone						
Young's modulus (GPa)	17.23 ± 2.71	15.92 ± 0.32	15.66 ± 3.29	17.95 ± 1.39	16.96 ± 0.76	18.18 ± 0.95
Hardness (GPa)	0.58 ± 0.10	0.54 ± 0.03	0.57 ± 0.18	0.60 ± 0.04	0.55 ± 0.03	0.65 ± 0.14

Trabecular bone						
Young's modulus (GPa)	14.88 ± 1.80	16.20 ± 1.32	14.99 ± 2.56	15.94 ± 1.75	16.88 ± 0.55	14.67 ± 0.76††
Hardness (GPa)	0.52 ± 0.05	0.57 ± 0.06	0.66 ± 0.14	0.52 ± 0.06	0.60 ± 0.05	0.60 ± 0.04

### 3.6 Cortical and trabecular bone loss occurred throughout disease mouse femora (6 weeks post-inoculation)

**Fig. 5:**
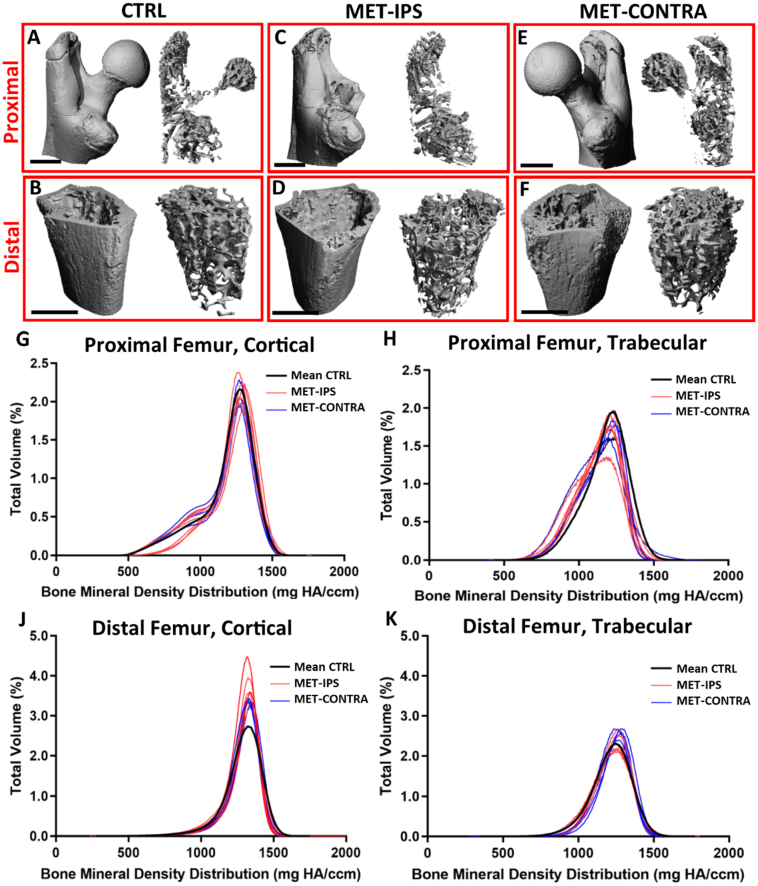
BALB/c disease and control mouse femurs at 6 weeks post-inoculation. 3D reconstructions of (A,C,E) proximal VOIs and (B,D,F) distal VOIs of a femur from each disease group, with contour isolation of (left) cortical and (right) trabecular bone regions. (G-K) Bone mineral density distribution (BMDD) curves in proximal and distal, cortical and trabecular regions of interest in BALB/c mouse femur VOIs at 3 weeks post-inoculation (CTRL *n* = 6, MET-IPS *n* = 7, MET-CONTRA *n* = 4).

**Fig. 6:**
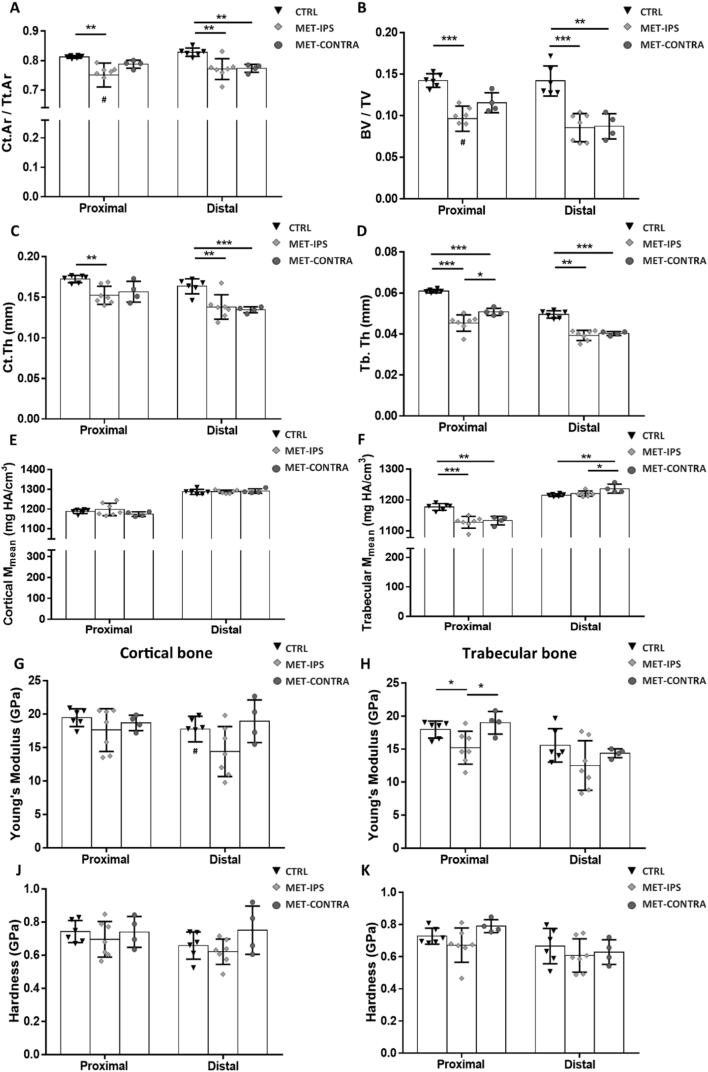
Mean parameters acquired from analyses of BALB/c mouse femurs 6 weeks post-inoculation. (A-F) Bone area fraction (Ct.Ar/Tt.Ar), bone volume fraction (BV/TV) cortical tissue thickness (Ct.Th), trabecular tissue thickness (Tb.Th) and mean mineral density (M_mean_) values acquired from micro-CT and BMDD analysis of proximal and distal VOIs in delineated cortical and trabecular bone regions. (G, H) Young's modulus and (J, K) hardness of cortical and trabecular bone tissue, obtained from nanoindentation tests in each region (CTRL *n* = 6, MET-IPS *n* = 7, MET-CONTRA *n* = 4). **#** Outlier, *p < 0.05, **p < 0.01, ***p < 0.001.

At 6 weeks post-inoculation, all MET-IPS and MET-CONTRA femurs formed osteolytic lesions in the greater trochanter, visible from 3D reconstructions of micro-CT scans (Fig. 5-C, 5-E). In 3 of 7 MET-IPS femur samples, the femoral head was entirely absent, with two of these samples also missing the femoral neck, while lesser and third trochanter bone tissue remained (Fig. 2-D). In the MET-CONTRA samples, 3 whole proximal regions were absent from femurs upon extraction. The working number of analysed MET-CONTRA femurs was therefore reduced to *n* = 4. All diaphysis and distal femur regions remained intact. Cortical bone area fraction (Ct.Ar/Tt.Ar) in the proximal femur, local to the tumour mass, was significantly lower in MET-IPS group (0.75 ± 0.04, *p* < 0.01) and MET-CONTRA group (0.79 ± 0.01 mm^2^, *p* < 0.05) compared to the CTRL group (0.81 ± 0.004) (Fig. 6-A). Similarly, Ct.Th in this proximal region was significantly lower in the MET-IPS group (0.15 ± 0.01 mm) and MET-CONTRA femurs (0.16 ± 0.11, *p* < 0.05) compared to the CTRL group (0.17 ± 0.004 mm, *p* = 0.001) (Fig. 6-C).

In the proximal femur trabecular region, BV/TV (Fig. 6-B) was significantly lower in the MET-IPS group (9.35 ± 2.0 %) and MET-CONTRA group (11.56 ± 1.03 %) compared to CTRL regions (14.22 ± 0.74 %) (*p* = 0.000, p < 0.05) at 6 weeks post-inoculation. Furthermore, M_mode_ was significantly lower (p < 0.05), and heterogeneity (FWHM) significantly higher (p < 0.001), in MET-IPS proximal trabecular femurs compared to CTRL results (Table 3). Interestingly, proximal femur trabecular bone M_mean_ was found to be lower in both MET-IPS (1127.67 ± 17.65 mg HA/cm^3^) and MET-CONTRA bone mineral (1133.22 ± 11.97 mg HA/cm^3^) compared to CTRL (1177.53 ± 9.89 mg HA/cm^3^, p = 0.000, *p* = 0.001), see Fig. 6-F. In the proximal femur region, Tb.Th was significantly lower in both the MET-IPS group (0.045 ± 0.004 mm) and MET-CONTRA group (0.051 ± 0.002 mm) compared to CTRL group (0.061 ± 0.001 mm) (p = 0.000, p = 0.000). Notably, proximal femur Tb.Th was also significantly lower in the MET-IPS femurs (0.045 ± 0.004 mm) compared to MET-CONTRA femurs from these same disease animals (0.051 ± 0.002 mm) (p < 0.05) (Fig. 6-D).

**Table 3:** Bone mineral content of healthy and disease mouse femurs 6 weeks post-inoculation (mean ± standard deviation) (CTRL n = 6, MET-IPS n = 7, MET-CONTRA n = 4) *p < 0.05, **p < 0.01, ***p < 0.001 relative to CTRL. †p < 0.05, ††p < 0.01, relative to MET-IPS.

	Units	Proximal Femur			Distal Femur		
		CTRL	MET-IPS	MET-CONTRA	CTRL	MET-IPS	MET-CONTRA
Cortical bone							
Tt.Ar	mm^2^	1.59 ± 0.06	1.24 ± 0.28*	1.44 ± 0.08*	0.58 ± 0.02	0.51 ± 0.04**	0.50 ± 0.02**
Ct.Ar	mm^2^	1.29 ± 0.05	0.94 ± 0.23**	1.13 ± 0.07*	0.48 ± 0.02	0.39 ± 0.05**	0.39 ± 0.02**
Ct.Ar/Tt.Ar	–	0.81 ± 0.004	0.75 ± 0.04**	0.79 ± 0.01*	0.83 ± 0.01	0.77 ± 0.03**	0.77 ± 0.01**
Ct.Th	mm	0.1723 ± 0.004	0.1523 ± 0.010**	0.1565 ± 0.011*	0.1650 ± 0.011	0.1378 ± 0.014**	0.1344 ± 0.003**
M_mean_	mg HA/cm^3^	1187.52 ± 9.76	1197.98 ± 29.10	1174.16 ± 10.27	1286.61 ± 11.64	1287.77 ± 7.52	1290.73 ± 10.15
M_mode_	mg HA/cm^3^	1276.29 ± 6.99	1283.66 ± 19.78	1277.79 ± 3.92	1330.22 ± 8.55	1331.17 ± 8.47	1302.34 ± 35.39
M low	%	2.58 ± 0.45	2.20 ± 1.41	3.00 ± 0.72	0.22 ± 0.06	0.22 ± 0.07	0.18 ± 0.04
M medium	%	88.16 ± 2.19	83.25 ± 6.21	83.26 ± 6.47	76.04 ± 15.15	80.00 ± 17.46	65.12 ± 17.44
M high	%	9.41 ± 2.26	14.54 ± 6.74	13.74 ± 7.11	23.75 ± 15.17	19.78 ± 17.51	34.69 ± 17.48
FWHM	mg HA/cm^3^	233.87 ± 16.84	229.32 ± 9.06	226.94 ± 16.65	192.55 ± 11.68	177.92 ± 12.35	185.66 ± 15.29

Trabecular bone							
TV	mm^3^	3.57 ± 0.15	4.35 ± 0.46**	4.47 ± 0.20**	3.21 ± 0.06	3.74 ± 0.33**	3.68 ± 0.23**
BV	mm^3^	0.51 ± 0.03	0.41 ± 0.11	0.52 ± 0.05	0.46 ± 0.05	0.32 ± 0.07**	0.32 ± 0.05*
BV/TV	–	0.14 ± 0.01	0.09 ± 0.02**	0.12 ± 0.01*	0.14 ± 0.02	0.09 ± 0.02***	0.09 ± 0.01**
M_mean_	mg HA/cm^3^	1177.53 ± 9.89	1127.67 ± 17.65***	1133.22 ± 11.97**	1215.72 ± 4.2	1220.38 ± 8.04	1236.51 ± 12.47*†
M_mode_	mg HA/cm^3^	1228.39 ± 6.62	1209.63 ± 14.05*	1224.16 ± 16.46	1247.99 ± 8.38	1257.21 ± 8.83	1269.69 ± 20.73
Conn.D.	mg HA/cm^3^	64.28 ± 8.31	83.02 ± 15.579	79.27 ± 9.17	140.75 ± 13.59	135.35 ± 40.75	141.63 ± 34.90
SMI	–	0.86 ± 0.03	1.38 ± 0.26***	1.13 ± 0.05**	1.48 ± 0.21	1.97 ± 0.31	2.11 ± 0.14**
Tb.N	1/mm	2.14 ± 0.15	2.14 ± 0.13	2.29 ± 0.29	3.86 ± 0.13	3.51 ± 0.57	3.71 ± 0.31
Tb.Th	mm	0.0611 ± 0.001	0.0454 ± 0.004***	0.0509 ± 0.002*** †	0.0496 ± 0.016	0.0393 ± 0.002**	0.0402 ± 0.001***
Tb.Sp	mm	0.49 ± 0.04	0.47 ± 0.03	0.45 ± 0.06	0.24 ± 0.01	0.29 ± 0.07*	0.26 ± 0.02
M low	%	0.54 ± 0.13	0.97 ± 0.46	1.18 ± 1.23	0.15 ± 0.06	0.12 ± 0.07	0.06 ± 0.02*
M medium	%	93.10 ± 5.62	93.49 ± 2.54	88.80 ± 5.30	94.73 ± 2.69	88.86 ± 9.69	78.81 ± 18.00
M high	%	6.36 ± 5.73	5.54 ± 2.41	10.02 ± 6.26	5.09 ± 2.75	11.01 ± 9.74	23.38 ± 16.14*
FWHM	mg HA/cm^3^	253.36 ± 24.77	341.28 ± 41.26**	316.14 ± 69.34	267.50 ± 19.50	282.05 ± 18.52	244.09 ± 9.27††

In the distal femur cortical bone, Ct.Th and Ct.Ar/Tt.Ar were significantly lower in the MET-IPS and MET-CONTRA groups compared to CTRL femurs (Fig. 6**-**C). Similarly, in distal femur trabecular regions, BV/TV and Tb.Th were also significantly lower in the MET-IPS and MET-CONTRA femurs compared to CTRL (Table 3). It is notable that distal trabecular mineral heterogeneity (FWHM) was significantly higher in the MET-IPS group (282.05 ± 18.52 mg HA/cm^3^) compared to the MET-CONTRA group (244.09 ± 9.27 mg HA/cm^3^, *p* < 0.01) but did not differ from CTRL samples (267.5 ± 19.5 mg HA/cm^3^, *p* = 0.231). Interestingly, low range trabecular bone mineral density (M_low_) had significantly decreased, and high range bone mineral density (M_high_) had significantly increased, in MET-CONTRA samples compared to CTRL samples at the 6 week time point, while no differences were seen in these ranges between tumour-adjacent MET-IPS and CTRL femurs (Fig. 5-K, Table 3).

### 3.7 Nano-mechanical properties lower in tumour-adjacent femurs (6 weeks post-inoculation)

At 6 weeks in the proximal region trabecular bone, Young's modulus in the MET-IPS group (15.21 ± 2.30 GPa) was found to be significantly lower when compared to both CTRL femurs (17.96 ± 1.17 GPa, *p* = 0.031) and to MET-CONTRA femurs (18.99 ± 1.48 GPa, *p* = 0.026), see Fig. 6-H. In the distal region, mean Young's modulus was significantly lower in the cortical bone of the MET-IPS femurs (14.39 ± 3.47 GPa) when compared to MET-CONTRA (18.92 ± 2.76 GPa, *p* < 0.05) (Fig. 6-G) but did not differ from CTRL (17.77 ± 1.75 GPa, *p* = 0.698). No significant differences in bone hardness were detected (Fig. 6-J, 6-K) (Table 4).

**Table 4:** Young's modulus and hardness (mean ± standard deviation) 6 weeks post-inoculation of 4T1 breast cancer cells, obtained from 10 nanoindentation mechanical tests in each region and bone tissue type of each femur. Includes mean ± standard deviation (CTRL *n* = 6, MET-IPS *n* = 7, MET-CONTRA *n* = 4). *p < 0.05, relative to CTRL. †p < 0.05 relative to MET-IPS.

	Proximal Femur			Distal Femur		
	CTRL	MET-IPS	MET-CONTRA	CTRL	MET-IPS	MET-CONTRA
Cortical bone						
Young's modulus (GPa)	19.46 ± 1.20	17.61 ± 2.96	18.68 ± 0.99	17.77 ± 1.75	14.39 ± 3.47*	18.92 ± 2.76*
Hardness (GPa)	0.74 ± 0.07	0.70 ± 0.01	0.74 ± 0.08	0.74 ± 0.05	0.67 ± 0.099	0.75 ± 0.13

Trabecular bone						
Young's modulus (GPa)	17.96 ± 1.17	15.21 ± 2.30*	18.99 ± 1.48†	15.56 ± 2.31	12.52 ± 3.47	14.37 ± 0.59
Hardness (GPa)	0.73 ± 0.05	0.67 ± 0.10	0.79 ± 0.04	0.67 ± 0.10	0.61 ± 0.10	0.63 ± 0.07

## 4 Discussion

This study reveals temporal changes in bone microarchitecture, mineral content and nano-mechanical properties local and distal to breast cancer metastatic tumours induced in an immunocompetent BALB/c mouse model inoculated with 4T1 breast cancer cells in the mammary fat pad. This is the first study to directly compare changes in bone tissue material properties upon breast cancer metastasis, both prior to and following the development of osteolytic lesions using the same immune competent animal model. In addition, our analyses were conducted in two distinct proximal and distal regions in femurs of both tumour-bearing and non-tumour-bearing long bones within the same disease animals, allowing for a comprehensive understanding of the impact of tumour presence on resulting changes in the bone mechanical environment. Moreover, thanks to the non-destructive nature of micro-CT, 3D bone mineral content analysis and mechanical testing was conducted on the same femur samples, which is not possible when utilising backscattered electron imaging (BSE) or Raman spectroscopy methods. The results from this study reveal no overt osteolytic destruction by 3 weeks post-inoculation, but trabecular thinning and increased bone mineralisation suggest early compensatory response to breast cancer metastatic invasion of bone tissue. Upon overt osteolytic destruction at the later time point of 6 weeks, significant decreases in bone mineral content and tissue properties occurred throughout both the ipsilateral and contralateral bones of the metastatic animals. These results reveal the time-dependant and spatial nature of changes in bone tissue, and specifically reveal that bone tissue composition is altered prior to the development of overt metastatic osteolysis, local and distant from the primary tumour site. Such changes observed in this study may arise either as a result of tumour-derived growth factors released upon the arrival of disseminated tumour cells, or might be a mechanobiological mineralisation response by bone cells in regions of elevated strain, as discussed in detail below.

Some limitations to this study require consideration. Firstly, skeletal responses to metastatic invasion may differ in the mouse model from human patients due to biological, anatomical, and musculoskeletal differences. However, mice exhibit similar bone morphological changes during ageing to humans (Jilka, 2013) and the 4T1-BALB/c mouse model consistently produces bone tissue metastasis and is not susceptible to the same degree of subject variation as arises in human studies. Secondly, only two time points were chosen for this study and later time points were not included, because by 6 weeks overt osteolysis was already established, and 9 weeks post-inoculation is reported to exceed the humane endpoint of mouse metastatic models due to high risk of fracture failure (Arrington et al., 2006). Thirdly, because methods required for histological analyses are destructive and impact bone properties (Currey et al., 1995, Nazarian et al., 2009), IVIS scans were used to confirm tumour cell presence in metastatic animals by 6 weeks in lieu of histology. However, our micro-CT imaging detected extensive osteolysis, providing further evidence of successful metastatic invasion. Moreover, H&E staining has confirmed the presence of metastatic tumour cells in femoral trabecular bone tissue at just 19 days in the same animal model (Lelekakis et al., 1999). Fourthly, dry nanoindentation was used in this study, which is reported to result in higher Young's modulus and hardness (compared to hydrated samples in C57BL/6 mice tibiae (Rodriguez-Florez et al., 2013). However, nanoindentation of dry bone tissue is suitable for comparative studies (Rho et al., 1999), as demonstrated in previous studies investigating patient and animal model breast cancer metastasis (Nazarian et al., 2008; Sekita et al., 2017). Testing of hydrated samples is limited to within 45 min of removal from storage in deionised water to avoid air drying effects (Rodriguez-Florez et al., 2013), which was not feasible given time taken for thermal drift to reach equilibrium (~1 h per batch test). An alternative method of maintaining sample hydration via a water bath setup is not suitable for a Berkovich indenting tip. It is important to note that all samples were tested dry, and so the differences reported between groups are valid. Finally, tumour masses on one side of metastatic mouse anatomy may cause asymmetric gait, whereby mice would offload the tumour-bearing hindlimb and increase weight on the contralateral limb out of discomfort, possibly altering bone physical properties in both femurs. While we did not quantify precise changes in limb loading throughout the animal study, no visible changes in mouse physical gait were noted through daily observational checks on these animals. It should be noted that femoral heads were absent for three metastatic ipsilateral and three contralateral animals by 6 weeks, which likely arose due to fractures during extraction of the femoral head from pelvic bone after osteolysis. However, such fragility did not arise in healthy control bones and thus this brittle behavior resulting in broken bone tissue, seen in both ipsilateral and contralateral femurs, is further evidence of the extent of bone loss in the metastatic animals. Had these femoral head regions remained intact, this may have revealed even greater changes in bone mineral content and nano-mechanical properties.

Breast cancer in late-stage patients undergoes metastatic spread to bone locations distant from the primary tumour, including the long bones, spine, pelvis, and ribs (Demers et al., 2000, Macedo et al., 2017). Key steps in the metastatic process are intravasation, circulation, evasion of host immune response, and subsequent extravasation of metastatic breast cancer cells from vasculature to arrive at the target tissue (Kretschmann and Welm, 2012). These processes are recapitulated in our immunocompetent mouse model in which a primary tumour was induced via mammary fat pad inoculation of breast cancer cells, and follows the metastatic cascade as would occur in vivo, rather than direct inoculation into the bone environment. This animal study has successfully replicated osteolysis due to breast cancer metastasis, as confirmed by micro-CT results in both tumour-adjacent ipsilateral femurs and contralateral femurs after 4T1 breast cancer cell inoculation. It is assumed that any evidence of metastatic invasion is a result of breast cancer cell extravasation from the vasculature within the bone microenvironment. Despite the development of tumour masses and evidence of heavy tumour burden at 3 weeks post-inoculation, micro-CT and bone mineral density distribution analysis revealed that overt osteolytic destruction was not detected until 6 weeks post-inoculation. However, trabecular thickness was reduced by 3 weeks and may indicate the initiation of osteolysis in the trabecular compartment. This finding is consistent with a study which reported breast cancer metastatic invasion was first detected in the trabecular bone marrow niche, 5 days after intracardiac or intravenous injection of cells into 12-week-old BALB/c mice (Allocca et al., 2019). Trabecular bone has a higher rate of bone turnover and metabolic activity compared to cortical bone tissue in patients (Clarke, 2008), and this higher bone metabolic activity is associated with higher rates of osteolytic destruction in BALB/c mouse models (Wang et al., 2015). BMD was similarly investigated in two studies involving MDA-MD-231 breast cancer cells directly injected into the distal femurs of female NCr nude mice (8–9 weeks old) (Arrington et al., 2006; Arrington et al., 2008). In both studies, osteolytic destruction was detected via radiography at 3 weeks post-injection but no significant changes in BMD were observed from micro-CT scans (10.46 μm^3^ and 12 μm^3^ voxel size, respectively), whereas by six weeks osteolytic tissue BMD was significantly lower compared to non-lesion and contralateral control femurs, and most animals did not reach the nine-week time point due to impending fracture. In a later study, 4-week-old BALB/c mice intravenously injected with MDA-MB-231 breast cancer cells were analysed for bone mineral content at 32 days, finding the bone volume fraction had decreased in metastatic trabecular bone of distal tibiae (9.5 ± 2.6 %) compared to healthy controls (22.7 ± 1.8 %) (Richert et al., 2015). Decreased cortical BMD (Kaneko et al., 2003) and trabecular bone volume fraction (Nazarian et al., 2008) have reported in patient studies of mixed sex and cancer type (breast, prostate, lung, colon) metastatic bone lesions located in femurs, far from primary tumours, such as those originating in breast tissue, but the time sequence of these changes have not been determined due to patient variation.

Our results reveal reduced bone tissue thickness and bone volume throughout tumour-bearing femurs, as well as decreased bone stiffness in proximal femur regions, when compared to healthy controls 6 weeks following primary tumour induction. In studies of human mixed cancer metastatic lesions, decreased compressive and tensile elastic modulus has been reported for cortical bone from the femur diaphysis (Kaneko et al., 2003) and decreased elastic modulus from dry nanoindentation tests has been reported in trabecular bone cores of the spine and femur (Nazarian et al., 2008). Though not as precise as analysis in larger bone specimens (human, rat long bones) due to limited scan resolution, micro-CT derived BMDD is utilised in the analysis of mouse femur bone tissue (Martín-Badosa et al., 2003; Ravoori et al., 2010; Bouxsein et al., 2010), and correlates closely with BSE imaging analysis in all instances except measurements for heterogeneity (full width at half maximum) (Mashiatulla et al., 2017). An animal study of female athymic rats (5–6 weeks old) receiving an intracardiac injection of HeLa osteolytic cancer cells reported significantly reduced bone mineral density (by BSE) in osteolytic lesions within lumbar vertebrae compared to healthy controls (Burke et al., 2017), which is in keeping with our findings upon osteolysis at 6 weeks. Interestingly, this study found no differences in Young's modulus or hardness by nanoindentation, citing mixed analysis of cortical and trabecular bone tissue locations to be a probable cause (Burke et al., 2017). Another animal study of C57BL/6 female mice inoculated with B16F10 cells via intracardiac injection (Sekita et al., 2017), reported reduced dry nanoindentation modulus in cortical bone from the femoral diaphysis after 14 days (metastatic: 18.3 ± 3.1GPa, control: 24.2 ± 2.3GPa). Large bone loss observed in distal tumour-bearing femur regions at 6 weeks post-inoculation may be explained by tumour mass growth as it expands distally over time, as indicated by bioluminescence of IVIS scans which extended to distal femur regions (Fig. 2**-**A). This is supported by an animal study of male SCID mice (8–10 weeks old) inoculated with prostate cancer cells (Ace1 or DU145) in the intramedullary cavity of the tibiae compared to saline-inoculated control mice (Sottnik et al., 2015), that reported increased intermedullary pressure exerted by a tumour mass, which induced osteocytes to secrete known promoters of prostate cancer metastasis in bone tissue (CCL5, MMPs). Alternatively, bone loss and reduced trabecular bone mineral density also occurred within the proximal and distal contralateral femurs at 6 weeks, which may be explained by systemic circulation of breast cancer cells during the migration step of the metastatic cascade (Langley and Fidler, 2007), resulting in the invasion of skeletal sites distant from the primary tumour, as seen in breast cancer metastatic patients (Demers et al., 2000, Macedo et al., 2017).

**Fig. 7:**
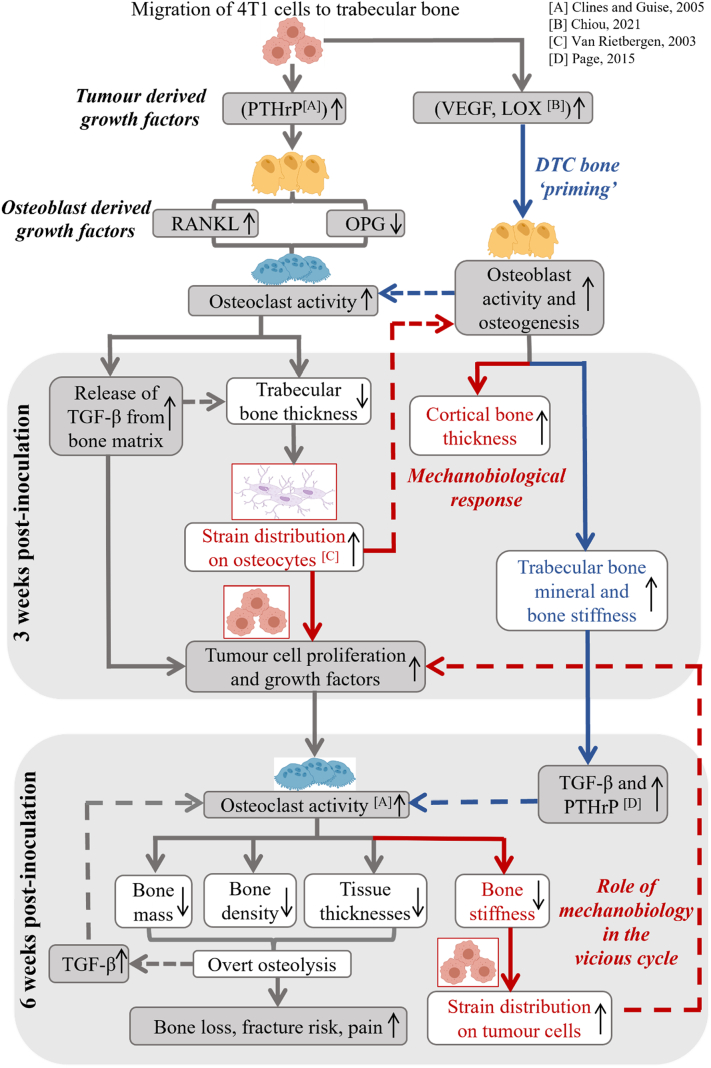
Proposed sequence of changes in bone microarchitecture and tissue composition during breast cancer metastatic invasion of bone tissue, 3 and 6 weeks after inoculation of breast cancer cells into the mammary fat pad. Growth factors released from 4T1 cells stimulate osteoclastogenesis and bone resorption. In addition to the known influence of growth factors released from the bone matrix in driving further tumour cell activity (Gray arrows), we report increased bone mineralisation at the early stages of osteolysis. We propose that these arise due to either, or both, of a) mechanobiologically driven responses by osteocytes to the altered mechanical environment following early osteolysis (Red arrows and textboxes) or b) bone niche ‘priming’ by factors produced by disseminated tumour cells (Blue arrows and textboxes) drive. These changes might lead to a secondary alteration in the mechanical environment of both the bone and tumour cells, driving further tumour cell proliferation and bone resorption, and thereby perpetuate the vicious cycle. Upward arrow, increase; downward arrow, decrease; dashed arrows, feedback mechanism; white boxes, results from this study. [A-D] References to relevant literature. Illustration made in ©BioRender - biorender.com.

Interestingly, by 3 weeks post-inoculation, there was an increase in bone mineralisation in the metastatic femurs compared to healthy bone tissue, along with increased distal femur cortical thickness. There are a number of possible explanations for these changes, which we consider herewith. Firstly, resorption of low bone mineral density in the superficial layers of trabecular bone might occur upon initiation of osteolysis and, because the centre of trabeculae are more highly mineralized (Brennan et al., 2011), the remaining trabeculae may have a higher density. Secondly, mineralisation could by stimulated by either the release of bone matrix proteins (OSC, OPN, Collagen type I) upon bone resorption (Cox and Morgan, 2013) or by tumour-derived growth factors released by disseminated tumour cells (Clines and Guise, 2005), both of which can stimulate resident osteoblasts to increase bone deposition. Interestingly, blood serum analysis from breast cancer metastatic patients found increases in bone matrix proteins occur only once osteolytic destruction is detected (Pollmann et al., 2007, Singhal et al., 1997, Suzuki et al., 1989), and so these may not explain the early increases in bone mineralisation reported here. A recent study reported increased trabecular and cortical bone thickness and evidence of bone formation after 3 weeks in animals that received daily intraperitoneal injections of tumour-cell conditioned media (Chiou et al., 2021). They proposed that tumour-derived growth factors (VEGF, Lysyl Oxidase) released by disseminated tumour cells (DTCs) act for the purpose of ‘priming’ the bone ECM to facilitate a more favourable microenvironment for DTC attraction, survival and proliferation (Chiou et al., 2021). VEGF is a known regulator of bone resorption (Zheng et al., 2013), while lysyl oxidase (LOX) release is driven by signaling of hypoxia inducible factors (HIF) to promote invading tumour cell colonisation and osteolysis at skeletal sites (Rankin et al., 2016). Thus, bone mineral priming, arising from hypoxic stress, might be responsible for the changes in tissue mineralisation reported here by 3 weeks. Alternatively, the early trabecular bone loss we identified may alter the normal distribution of mechanical strain within the bone and thus initiate a mechanobiological mineralisation response by the bone cells residing in regions of elevated strain. Indeed, decreased trabecular bone arising during osteoporosis has been shown to lead to elevated strain on the cortical bone of the proximal femoral neck (Van Rietbergen et al., 2003, Verhulp et al., 2008). Alteration of the mechanical environment may also dictate the production of growth factors by the tumour cells and tumour cell activity. An in vitro study introduced MDA-MB-231 to culture medium from osteoclasts, previously conditioned in the media of osteocytes (MLO-Y4) subjected to 2 h of oscillatory fluid flow stimulation (Ma et al., 2018). This study reported reduced migration and increased apoptosis of these breast cancer cells, compared to non-mechanically stimulated osteocyte conditioned media (Ma et al., 2018). These findings are reflected in previous human and animal studies where exercise or direct mechanical loading regimes of long bones inhibit tumour progression, proposedly due to altered TGF-β signaling and sclerostin secretion in mechanosensitive osteocytes (Sarazin et al., 2021). Notably, a study of MDA-MB231 breast cancer cell-inoculated proximal tibiae, in SCID mice subjected to dynamic compressive loads for 6 weeks, reported inhibited osteolytic progression, via limited loss of bone volume and trabecular thickness, during metastasis suggested to be a result of mechanoregulation of osteoblast and osteoclast activity (Lynch et al., 2013). Interestingly, increased matrix rigidity has been shown, in vitro, to induce more active tumour invasion and osteolytic destruction of MDA-MB-231 breast cancer cells, via increased integrin β3 mediated expression of TGF-β and PTHrP (Page et al., 2015), and demonstrates the important role of mechanobiology in breast cancer metastatic invasion. Mechanical stimulation has also been reported to stimulate breast cancer cellular behavior in the context of primary tumour development and cancer cell extravasation. In particular, increased bone marrow ECM interstitial fluid flow, hydrostatic pressure, tissue strain and ECM stiffness, known to actively drive resident bone cellular activity and remodelling, are suggested to provide mechanical cues which drive tumour malignancy and cancer cell extravasation (Lynch et al., 2020). In this way the mechanical environment might also play a key role in stimulating both tumour and bone cell activity and contribute to tumour cell behavior during the cancer vicious cycle. Elevated bone stiffness by three weeks post-inoculation in this study, in only the distal ipsilateral femur regions, may be due to increased load-bearing upon the introduction of a tumour mass. However, the precise impact of mammary pad-inoculated tumour weight on murine bone mineral content and mechanical properties is not yet known. Fig. 7 illustrates how both proposed mechanisms may simultaneously act to drive the temporal changes in bone tissue that we observe here. These changes may alter the tumour-adjacent and non-tumour-bearing mechanical environments of bone and breast cancer metastatic cells over time, and highlights the potential role of mechanobiology in perpetuating tumour cell proliferation during the cancer vicious cycle and tumour invasiveness during breast cancer metastasis to bone. Further studies are required to delineate whether DTC ‘priming’ of the bone metastatic niche, or a mechanobiology-driven response to imbalanced strain distributions in the bone ECM, would elucidate these changes.

## 5 Conclusion

Temporal and spatial analysis of bone physical properties upon breast cancer cell metastatic invasion provides an understanding of the changes in bone microarchitecture and tissue composition. Comprehensive analysis of bone mineralisation and nano-mechanical properties at 3 weeks post-inoculation indicates early bone tissue changes in response to breast cancer metastatic invasion. In the longer term, decreased mineral content and lower bone tissue stiffness in tumour-loaded femurs occurred upon osteolytic destruction. These changes may alter the mechanical environment of both the bone and tumour cells, and thereby play a role in perpetuating the cancer vicious cycle during breast cancer metastasis to bone tissue.

## CRediT authorship contribution statement

**Anneke S.K. Verbruggen:** Conceptualization, Methodology, Formal analysis, Investigation, Writing – original draft, Writing – review & editing, Visualization. **Elan C. McCarthy:** Methodology, Visualization. **Roisin M. Dwyer:** Methodology, Resources, Writing – original draft, Project administration, Funding acquisition. **Laoise M. McNamara:** Conceptualization, Project administration, Writing – original draft, Writing – review & editing, Supervision, Funding acquisition.

## Declaration of competing interest

None.
